# Recognition of delirium in ICU patients: a diagnostic study of the NEECHAM confusion scale in ICU patients

**DOI:** 10.1186/1472-6955-4-7

**Published:** 2005-12-13

**Authors:** Henny EM Immers, Marieke J Schuurmans, Jaap J van de Bijl

**Affiliations:** 1 Intensive Care Unit, Reinier de Graaf Group, Reinier de Graafweg 3, 2625 AD Delft, the Netherlands; 2 Current address: CC department, Haga Hospital, location Leyenburg, Leyweg 275, 2545 CH The Hague, the Netherlands; 3 Nursing Sciences, Julius Center, Stratenum, University Medical Center Utrecht, Heidelberglaan 100, 3584 CX Utrecht, the Netherlands; 4 Faculty of Health Care, University of Professional Education, Bolognalaan 101, 3484 CJ Utrecht, The Netherlands

## Abstract

**Background:**

A delirium, is a serious, high-frequency complication in intensive care unit (ICU) patients. The consequences of this complication range from high morbidity and mortality to greater need for nursing care. Despite these, delirium is often not recognized and there for not treated. In this study a nursing screening instrument, the NEECHAM confusion scale, was studied for early recognition of delirium ICU patients. This scale proved valid and reliable in several studies in the general hospital population.

**Methods:**

In this study validity and reliability were tested in a prospective cohort of 105 patients. Gold standard for delirium was an independent DSM-IV diagnosis. User friendliness was tested by structured evaluation of nurses' experiences working with the scale.

**Results:**

The NEECHAM confusion scale showed high internal consistency (Cronbach's alpha 0.88) and an interrater reliability of Cohen's Kappa 0.60. The concurrent validity with the DSM-IVcriteria showed a strong link (chi-square 67.52, p [less than or equal to] 0.001). Sensitivity was high, 97% and specificity was good 83%. ICU nurses completed the NEECHAM confusion rating in 3.69, ± 1.21 minutes average. In general the nurses were positive about the NEECHAM confusion scale. They were able to collect data during regular care, but experienced problems in rating the scale in intubated patients. The items in themselves were clear, the content validity, measured by the language used was rated good.

**Conclusion:**

The psychometric characteristics of the NEECHAM confusion scale of this ICU study are generally consistent with validity research previously reported for the general hospital population. The psychometric characteristics and the ease of use of the NEECHAM confusion scale enables ICU nurses to early recognize delirium. Further study, especially in intubed patients is recommended.

## Background

Delirium is a serious, high frequency complication in intensive care unit (ICU) patients (incidence: 40–82%) [1-5], being defined as a transient organic mental syndrome characterised by a disturbance in awareness, cognition and attention [6]. These disturbances are frequently manifested in expressions of disorientation, memory impairment, disturbance in mental processes, motor unrest, sleep problems, anxiety and agitation. Abrupt onset and fluctuation of the symptoms are characteristic, and onset frequently occurs from within a few hours to a few days of hospital admission. Symptoms of delirium can present differently: in a hyperactive/hyper-alert form, a hypoactive/hypo-alert form, or a combination of these two [7]. The patient can be restless, agitated and aggressive. In the delirious state, the patient will often remove his or her lines, catheters and/or tube. Nurses are most familiar with the hyperactive/hyper-alert form and often overlook the hypoactive/hypo-alert type of patients [1], who are calm and sleepy, react slowly and seldom move. Sometimes they mumble to themselves or make inappropriate gestures. Research shows that >60% of all cases delirious patients, [8] go unrecognised by doctors and nurses. Systematic screening of cognitive functions is rare in general hospital practice [1] and a delirium is often not considered a potential medical emergency. Moreover, in elderly patients delirium symptoms are often mistaken for symptoms of dementia or another mental illness [9,10]. Therefore these patients are treated either inadequately or not at all [2,11,12]. The consequences for the patient can be serious, entailing greater risk of morbidity and mortality, longer hospital stays, greater need for nursing care, and greater risk of complications, bodily injuries in general, and more frequent admission to nursing homes following discharge from hospital [1,5,13]. The high prevalence and the severity of the consequences emphasises the fact that delirium should be a major concern for ICU staff [8].

Nursing staff monitor the patient 24 h a day; with little effort they can enable early recognition of a delirium, and enhance the prognosis and benefits of any treatment [1,14,15]. To achieve this, there is a need to increase awareness, and to develop and implement valid, user-friendly assessment tools [4].

A nursing observation scale was tested for use in ICU patients. This scale, the NEECHAM confusion scale, proved valid and reliable in several studies in the general hospital population [16-18,21,22]. A small pilot study in 19 ICU patients [19] gave positive results.

The aim of our study was to extend testing of the NEECHAM confusion scale for use in ICU patients by determining its reliability, validity and user-friendliness in monitoring this group of patients.

## Methods

### Design and sample

The reliability, validity and user-friendliness of the NEECHAM confusion scale were tested in a prospective study. Following approval from the Medical Ethics Committee the study, was conducted in the Intensive Care Unit (9 beds) of a medium-sized general hospital.

Patients were included if they had given informed consent, were present at the time the ICU nurses completed the scale, were not under the influence of barbiturates and/or sedatives, and were able to be interviewed (i.e. they were not too seriously ill, and were Dutch or English speaking). Of 105 patients, 45 were women (43%) and 60 men (57%). The patients stayed in the ICU between 1 and 14 days (on average 2.34, ± 3.6 days). The average age of the patients was 68.3 ± 13.5 years.

All ICU nurses (n = 39) in the unit were asked to participate in the study. Responses on the user-friendliness of the scale were collected from 36 nurses (92%), 26 women (72%) and 10 men (28%). The average age of the ICU nurses was 36.5 ± 7.9 years,. The number of years of work experience of the nurses in the ICU unit was on average 9.4 ± 7.4 years.

#### Procedures

Preceding the study, all nurses were trained in rating the NEECHAM confusion scale. On weekdays during the routine 4 pm rounds, the ICU nurses rated the NEECHAM confusion scale in each patient who was participating in the study. A total of 253 ratings of patients were made, of which almost half were seen immediately after the nurses had completed the NEECHAM confusion scale (123 ratings in 53 patients) by a psychiatric intern who, blinded from the nurses' ratings, assessed and diagnosed the behaviour of the patient. The Diagnostic Statistical Manual of Mental Disorders (DSM)-IV criteria for delirium were used as the gold standard for the diagnosis. The 123 completed NEECHAM ratings and the interns' DSM-IV-TR diagnosis were used to define the diagnostic value of the NEECHAM scores.

In addition, one of the researchers (HI, ICU nurse) independently rated the NEECHAM confusion scale on a number of different days, 10 – 15 min after the ratings of the ICU nurse. A total of 39 completed NEECHAM ratings were used to determine the interrater reliability.

At the end of the study, the ICU nurses were asked to complete a questionnaire on the user-friendliness of the scale. The questionnaire contained 18 questions to be rated on a 5-point Likert scale and one open question. Questions were formulated based on a literature review on requirements of a delirium screening scale that need to be met for successful implementation in the ICU [20].

#### The NEECHAM confusion scale

The NEECHAM confusion scale, developed by Neelon and Champagne et al. [17], measures cognitive dysfunction such as delirium. This scale was extensively tested in the general hospital population [16-19,21,22] and is based on observations made by nurses during the routine rounds at the bedside. It measures delirium in the early stages and can be easily administered several times, since it takes only a few minutes to complete. It is not burdensome for the seriously ill patient.

The scale consists of 9 items divided over 3 subscales. Each item consists of 3 to 6 descriptions. Subscale 1 (information processing) measures attention, processing commands and orientation; subscale 2 (behaviour) measures appearance, motor and verbal behavior; Subscale 3 (physiological condition) measures vital function, oxygen saturation and urinary continence. The descriptions of the item verbal behaviour refer to speaking, ability to have a conversation. Intubated patients who are not sedated can often communicate only non-verbally. We included intubated patients in this study. Inclusion of intubated patients was described as one of the requirements that needed to be met for successful implementation in the ICU [20]. In the questionnaire on user-friendliness this aspect was evaluated.

The overall score of the NEECHAM ranges from 0 through 30 points. A score of 30 indicates that the patient gives a maximal (normal) reaction and 0 indicates a minimal reaction. The scale gives four grades of outcome: moderate to severe confusion and/or delirium (0–19 points), mild to early confusion and/or delirium(20–24 points), 'not confused' but at high risk of confusion and/or delirium (25–26 points), and normal cognitive functioning i.e. absence of confusion and/or delirium (27–30 points).

#### Statistical analysis

The reliability of the NEECHAM confusion scale was determined by means of the following reliability indicators: interrater reliability (Cohen's Kappa) and internal consistency (Pearson's item-total coefficient of correlation, Pearson's inter-item coefficient of correlation and Cronbach's alpha).

The validity of the NEECHAM confusion scale was determined by the following measures of validity: concurrent validity (chi-square) with the DSM-IV-TR diagnosis, construct validity (principal component analysis), and diagnostic value (sensitivity and specificity).

The user-friendliness was determined by calculating the average score per question.

All data were processed using SPSS.

## Results

An overall score of ≤ 19 points on the NEECHAM, indicating moderate to severe acute confusion and/or delirium, was found in 49 (19.4%) ratings. An overall score of between 20 and 24, indicating mild or early acute confusion and/or delirium, was found in 40 (15.8%) ratings.

All other ratings (n = 164, 64.8%) with scores of 25 or higher, indicated normal cognitive functioning. In 72 (43.9%) of these, a NEECHAM score of 25 or 26 was found. This score indicates non-acute confusion and/or delirium, however, and the presence of high risk.

### Reliability

The NEECHAM Confusion Scale showed high internal consistency in this study (Chronbach's alpha 0.88). The item-total correlation (Table 1) correlated well with information processing (attention, processing of commands, orientation) and behaviour (appearance, motor, verbal) (Pearson r = 0.68), while the urinary continence control (Pearson r 0.24) and vital function/oxygen saturation (Pearson r = 0.20) correlated weakly or not at all with the item-total coefficient of correlation. Table 1 shows under 'alpha if item deleted' that removal of these items increases the alpha values.

**Table 1:** Pearson item-total coefficient of correlation of the NEECHAM confusion scale (n = 253)

Alpha if item Deleted	Correct Item- total Correlation	
Attention	.8644	.8392
Processing commands	.8815	.8346
Orientation	.8698	.8361
Appearance	.6821	.8627
Motor	.8235	.8425
Verbal	.7948	.8456
Vital function	.1158	.8983
Oxygen sat.	.1956	.8887
Continence.	.2444	.8888

The correlation between the items ranges from 0.04 to 0.90 (Table 2); a low correlation was found between vital function (Pearson r 0.04 to 0.06) and oxygen saturation (Pearson r 0.09 to 0.27) and the other items. A fair correlation was found between urinary continence (Pearson r 0.13 to 0.32) and all the other items, which in turn correlated strongly with each other (Pearson r 0.61 to 0.90). The interrater's reliability indicated a reasonable conformity between raters above the expected coincidental conformity with Cohen's Kappa of 0.60.

**Table 2:** Pearson Inter-item coefficients of correlation of the NEECHAM confusion scale (n = 253)

Items Continence function	Attention	Processing Commands	Orientation	Appearance	Motor	Verbal	Vital funct.	Oxygen Sat.	
Attention	1.0000								
Processing commands	.9007	1.0000							
Orientation	.8665	.8954	1.0000						
Appearance	.6465	.6646	.6613	1.0000					
Motor	.8353	.8615	.8133	.6150	1.0000				
Verbal	.7412	.7943	.7899	.6174	.7416	1.0000			
Vital function	.0618	.0479	.0506	.0461	.0363	.0504	1.0000		
Oxygen sat.	.0900	.1077	.1365	.1365	.0685	.1679	.2720	1.0000	
Continence	.1741	.1282	.1698	.1698	.1604	.1741	.3190	.2989	1,000

### Construct validity

A principal component analysis (Table 3) was performed to study in more detail the inter item correlations. After varimax rotation, two eigenvalues greater than 1 (4.9 and 1.5) were found, suggesting that two sources contribute considerably to the distributed variation among the items. Together, Components 1 and 2 account for 54.6% and 17% respectively, i.e. 71.7% of the variation in the 9 variables. The attention, processing commands, orientation, appearance, motor and verbal items load from fairly high (0.76) to high (0.96) on the first component. Vital function, oxygen saturation and urine continence score fairly high (0.71 – 0.74) on the second component.

**Table 3:** NEECHAM confusion scale Component Loading and Communalities (n = 253)

NEECHAM Item Communality	Component 1		Component 2
Attention	0.930	6.313E-02	0.868
Processing commands	0.955	4.131E-02	0.913
Orientation	0.935	8.002E-02	0.880
Appearance	0.762	0.121	0.596
Motor	0.909	3.392E-02	0.827
Verbal	0.864	0.110	0.759
Vital function	-1.715E-02	0.738	0.545
Oxygen saturation	7.478E-2	0.707	0.506
Urine continence	0.134	0.731	0.553

Eigenvalue	4.912	1.534	
% explanatory var.	54.573	17.048	

* Rotation method: Varimax matrix

### Concurrent validity

The concurrent validity with the DSM-IV-TR criteria showed a strong link (chi-square 67.52, p = 0.001). The diagnostic value of the scale can be considered fairly high to high with a sensitivity of 97.2% and a specificity of 82.8% (Table 4).

**Table 4:** Clinical index to predict delirium diagnosed according to the DSM-IVcriteria by ratings of the NEECHAM confusion scale* (n = 123)

Sensitivity [(35/36) × 100]	97.2%
Specificity [(72/87) × 100]	82.8%
Predicted value for positive test [(35/50) × 100]	70.0%
Predicted value for negative test [(72/73) × 100]	98.6%
Precision [(35+72/35+15+1+72) × 100]	86.9%

* NEECHAM ratings of 0–24 were rated as delirious

### User-friendliness

ICU nurses completed the NEECHAM ratings in 3.69 minutes (SD 1.21) average. In general the nurses were positive about the NEECHAM confusion scale (Table 5). They were able to collect the data during regular care, but experienced problems in rating the items orientation and verbal behaviour in intubated patients. The items were in themselves clear for all nurses; with proper instructions, their experience was that, in general, the rating scale gave no problems. Content validity, measured by the language used, was rated good. Completing the ratings with intubated patients (5% of all included patients were intubated and not under influence of barbiturates and/or sedatives) presented some problems. One can rate these items based on non-verbal communication, although this option is not explicitly given on the scale. Some nurses also had doubts regarding the rating of item appearance (item 4), motor behaviour (item 5) and the measurement of the vital signs (item 7, 8, 9). Doubts regarding the item 4 arise because ICU patients are usually unable to take care of their own appearance. Doubts regarding motor behaviour arose because ICU patients frequently have polyneuropathy, which can influence the rating of motor behaviour. The value of rating the physiological items was questioned since these are disturbed in most ICU patients.

**Table 5:** User-friendliness of the NEECHAM confusion scale (n= 36)

Questions and statements *	Mean ratings
How much time did you need to rate the NEECHAM confusion scale? (in minutes)	3.69 (1.21)
All observational data were collected during regular bedside care	4.08 (1.21)
Collection of data for rating the NEECHAM confusion scale did not ask specific effort of the patient	3.53 (1.32)
I can assess the patient for all items of the NEECHAM confusion scale	3.33 (1.37)
I can assess intubated ICU patients for all items of the NEECHAM confusion scale	2.11 (0.89)
I can assess ICU patients with a level of awareness comparable with an EMV score of 3 - 5- 3 or higher for all items of the NEECHAM confusion scale	3.42 (0.81)
Item 1 of the NEECHAM confusion scale is clearly formulated	4.06 (0.58)
Item 2 of the NEECHAM confusion scale is clearly formulated	4.08 (0.50)
Item 3 of the NEECHAM confusion scale is clearly formulated	4.00 (0.68)
Item 4 of the NEECHAM confusion scale is clearly formulated	3.78 (0.90)
Item 5 of the NEECHAM confusion scale is clearly formulated	3.92 (0.69)
Item 6 of the NEECHAM confusion scale is clearly formulated	3.97 (0.81)
Item 7 of the NEECHAM confusion scale is clearly formulated	4.22 (0.48)
Item 8 of the NEECHAM confusion scale is clearly formulated	4.22 (0.48)
Item 9 of the NEECHAM confusion scale is clearly formulated	4.19 (0.58)
With the instructions, one can rate the NEECHAM confusion scale	4.14 (0.42)
The language used in the NEECHAM confusion scale is appropriate	3.69 (1.06)
All the items in the NEECHAM confusion scale are relevant for the ICU patient	3.42 (1.05)

Provide any points of particular interest where the NEECHAM confusion scale could be improved with regard to user friendliness that heave not yet been dealt with * statements were rated on a 5 point Likert scale: 1 = strongly disagree, 2 = disagree, 3 = neither agree nor disagree, 4 = agree, 5 = strongly agree

The ICU nurses gave valuable responses to the open question with regard to possible improvements to the scale. For example, with item 8 of the NEECHAM (oxygen saturation), the nurses found that the normal value (93 or higher, according to the NEECHAM confusion scale) for a COPD (chronic obstructive pulmonary diseases) patient was too high. Frequently, the normal O_2 _saturation value for this patient group in the ICU unit is taken to be between 90 and 92. With regard to the lay-out of the scale they suggested changes to improve the user-friendliness. In this study, the scale was printed on two pages, but the nurses preferred a denser format in which the scale could be completed on one page.

## Discussion

The psychometric characteristics of the NEECHAM confusion scale of this ICU study are generally consistent with validity research previously reported for the general hospital population. The interrater's reliability between two raters (kappa 0.60) is, as in Neelon's research [16] (0.65), fairly good. The internal consistency of the scale is good, as it is in all the other studies [16-19] (Cronbach's alpha ≥ 0.81), and the physiological functions did not correlate with the total item coefficient of correlation. On the basis of both the results from the internal consistency and from the principal component analysis (PCA), one might suggest that the vital functions and oxygen saturation be removed from the NEECHAM confusion scale. Like the studies of Neelon [16] and Milisen [18], two components were found after a PCA. Neelon [16], however, divided the instrument into three subscales, which appears to imply that there are three components.

The chi-square value of 67.5 (p = 0.001) between the DSM-IV-TR criteria and the NEECHAM scale show good concurrent validity. The sensitivity is just as high (> 90%) as in other studies; however, we found the specificity of 82.8% to be higher in this study than in that of Neelon (78%) [21].

The user-friendliness of the NEECHAM scale has not been previously examined in this way. The ICU nurses rated the user-friendliness generally as good. However, the scale in its present form is not suitable for use with intubated ICU patient. Adjustment of two items for non-verbal communication should be further studied.

Possible limitations of the present study could be the reliability of the gold standard (diagnosis by a psychiatric intern), and the collection of the data by nurses during regular care. Both of these elements, however, are representative of clinical reality and therefore enhance the implementation of the results.

## Conclusion

We conclude that the NEECHAM confusion scale has good psychometric characteristics and is easy to use with ICU patients who are not being intubated. In view of the high diagnostic value of the scale, the instrument is promising for screening delirium in ICU. Further study of the scale is needed to adapt it for use with intubated patients. The results of the PCA in this study suggest that the items with regard to the physiological condition could be removed. This would give a reduction of the scale from nine to six items which would enhance its user-friendliness, but more work is needed to confirm this conclusion.

Delirium in ICU patients is a serious and frequent adverse event. Current ICU clinical practice does not routinely screen for behavioural changes, as a result of which many delirious patients are not recognised in time. The NEECHAM confusion scale offers an opportunity to screen behaviour with little stress on either the seriously ill ICU patients or the busy ICU nurses. The instrument is simple, repeatable, and quick to apply on the basis of observation during regular care. The ready availability of an assessment tool is the first step in enhancing the quality of care for this group of patients. Improvement in detection methods and increasing knowledge, however, is not sufficient. To ensure use of these methods not only requires the requisite knowledge, but also acknowledgement of the emergency that a delirium can present [9].

## Competing interests

The author(s) declare that they have no competing interests.

## Authors' contributions

MS proposed the original idea for the study and HI, MS and JB designed the study protocol. HI executed the study and the statistical analyses. MS and JB were involved in the interpretation of data. HI drafted the manuscript, MS and JB read the manuscript critically for important intellectual content. MS prepared the international publication. All authors read and approved the final manuscript.

## Pre-publication history

The pre-publication history for this paper can be accessed here:


